# Primary intestinal lymphangiectasia: French National Diagnosis and Care Protocol (PNDS; Protocole National de Diagnostic et de Soins)

**DOI:** 10.1186/s13023-025-03657-9

**Published:** 2025-07-10

**Authors:** Stéphane Vignes, Mathieu Uzzan, Lionel Arrivé, Françoise Le Pimpec-Barthes, Claire Fieschi, Guillaume Lefèvre, Manon Javalet, Joanna Bettolo, Béatrice Dubern

**Affiliations:** 1 Department of Lymphology, Referral Center for Primary Lymphedema, Cognacq-Jay Hospital, 15, rue Eugène-Millon, 75015 Paris, France; 2 Department of Gastroenterology, Henri-Mondor Hospital, 1 rue Gustave Eiffel, 94000 Créteil, France; 3 Department of Radiology, Hôpital Saint-Antoine, 184, rue du Fg Saint-Antoine, 75012 Paris, France; 4 Department of Thoracic Surgery, HEGP, 20 rue Leblanc, 75015 Paris, France; 5 Department of Immuno-Hematology, Saint-Louis Hospital, 1, av. Claude-Vellefaux, 75010 Paris, France; 6 Institut of Immunology, CHU de Lille, bd du Pr Jules-Leclercq, 59037 Lille, France; 7 Department of Pediatric Gastroenterology and Nutrition, Armand-Trousseau Hospital, 26, avenue du Docteur Arnold-Netter, 75012 Paris, France

**Keywords:** Primary intestinal lymphangiectasia, Waldmann’s disease, Diagnosis, Explorations, Complications, Treatment

## Abstract

**Background:**

Primary intestinal lymphangiectasia or Waldmann’s disease (ORPHA code: 90362) is a very rare disorder of unknown etiology, characterized by digestive lymphatic vessel dilations.

**Main body of the abstract:**

The objective of the French National Diagnosis and Care Protocol (PNDS; Protocole National de Diagnostic et de Soins) is to provide health professionals with information about the optimal management and care for patients, based on a critical literature review and multidisciplinary expert consensus. The PNDS, written by members of the French National Reference Centers for Rare Vascular diseases and Rare Digestive diseases, is available from the French Health Authority website. The latter allow lymph leakage (chyle) into the intestinal lumen that is responsible for protein-losing gastroenteropathy, combining hypoalbuminemia, lymphopenia and hypogammaglobulinemia. Diagnosis is usually made before the age of 3, but primary intestinal lymphangiectasia may be discovered in adults. Edema of the lower limbs is the main clinical sign and serous effusions (pleura, peritoneum, pericardium) are sometimes abundant. Exudative gastroenteropathy is confirmed by increased α_1_-antitrypsin clearance. Esophagogastroduodenoscopy finds milky lesions corresponding to lymphangiectasias; duodenal biopsies confirm the diagnosis. Endoscopic videocapsule may be useful to evaluate the extent of the disease and/or if esophagogastroduodenoscopy is not contributory. In rare cases, the disease may be complicated by digestive or extra-digestive B-cell lymphoma in adults. Management is mainly based on a strict fat-free diet, combined with supplementation with medium-chain triglycerides, essential fatty acids and fat-soluble vitamins. Octreotide, a somatostatin analogue, has inconsistent efficacy, in combination with the fat-free diet and the sometimes-prescribed mammalian target of rapamycin-receptor inhibitor sirolimus, occasionally achieving positive effects. Diuretics and albumin infusions may be useful in addition to the fat-free diet. Intestinal resections are proposed for rare, localized, segmental forms of the disease.

**Short conclusion:**

Primary intestinal lymphangiectasia is a chronic disease requiring a prolonged restrictive and constraining strict low-fat diet supplemented with medium-chain triglycerides and fat-soluble vitamins. Its evolution can be complicated by more-or-less severe serous effusions and rare lymphoma. Life-long clinical and biological monitoring is required.

**Supplementary Information:**

The online version contains supplementary material available at 10.1186/s13023-025-03657-9.

## Introduction: Definition of the disease

Waldmann initially described primary intestinal lymphangiectasia (PIL) also called Waldmann’s disease, in 1961 based on 18 patients [1]. All patients had edema, hypoalbuminemia and hypogammaglobulinemia. He was able to demonstrate digestive leakage of albumin, while biopsies of the small intestine showed more-or-less marked dilations of the mucosal and submucosal lymphatic vessels, which he called “intestinal lymphangiectasias”. They are considered primary after elimination of the diseases causing secondary lymphangiectasias.

The objective of this French National Diagnosis and Care (PNDS) Protocol is to explain, to the medical and paramedical professionals involved, the current optimal diagnostic and therapeutic management, and care pathway of a patient with PIL. It aims to optimize and harmonize the management and follow-up of this rare disease throughout the country. It also identifies the specialties involved, an indication not provided in the Marketing Authorization, as well as the specialties, products or services necessary for the strict dietary needs of patients but not usually covered by the French National Health Insurance (FNHI). This PNDS can serve as a reference for the treating physician (specialists and general practitioners associated with FNHI) in consultation with the specialist physician, particularly when establishing the care protocol jointly with the experts at the Vascular Diseases Referral Center or the Rare Digestive Diseases Referral Center. However, the PNDS does not consider all specific cases, all comorbidities or complications, all therapeutic particularities, all hospital-care protocols, etc. It cannot claim to be a complete list of all cases, nor exhaustivity of possible management behaviors. Moreover, it cannot replace each physician’s direct responsibility to the patient. However, the protocol does describe the gold standard management of a patient with PIL. It should be updated as new data are validated.

This PNDS was developed according to the "Method of formulating of a national protocol of diagnosis and care for rare diseases" published by the Haute Autorité de Santé (HAS, Superior Health Authority) in 2012 (the methodological guide available at its website: www.has-sante.fr).

## Etiology and pathogenesis

PIL etiology remains unknown at present. Abnormalities in the regulation of genes involved in lymphatic system development have been reported: vascular endothelial growth factor recepto-3 (*VEGFR3*), prospero-related homeobox-transcriptional factor-1 (*PROX1*), forkhead/fox transcriptional factor-C2 (*FOXC2*), sex-determining region Y-box transcription factor-18 (*SOX18*) [2]. Mutations in the hepatocyte growth factor/mesenchymal–epithelial transition factor (*HGF/MET*) gene could also be involved in PIL [3].

PIL leads to exudative enteropathy with plasma leakage into the digestive tract lumen, with loss of plasma proteins being the main cause of edema. The decreased plasma-protein concentration is not directly related to their digestive loss but rather to insufficient synthesis unable to compensate for the protein degradation in the intestinal lumen. Long half-life proteins, such as albumin or immunoglobulin (Ig) G, are primarily affected. Plasma levels of shorter half-life proteins, e.g., prealbumin, fibrinogen and/or IgA, IgM and IgE, are usually normal or only slightly diminished [4]. Lymphatic system abnormalities result in losses of T cells and chylomicron-rich lymph—chyle—engendering lymphopenia, hypocholesterolemia, essential fatty acids (EFA) deficiency and steatorrhea.

## Epidemiology

PIL is very rare, although its exact prevalence remains unknown, particularly because of the existence of a- or pauci-symptomatic forms. It usually begins in childhood, before 3 years, or in young adults, but can sometimes be diagnosed in adults [5, 6]. Very rare familial forms also exist [1, 7] that are more frequent in association with certain syndromes, notably: RASopathies (Noonan, ORPHA:648, protein tyrosine phosphatase non-receptor type-11 (*PTPN11*, rat-sarcoma GTPase (Ras)-like without CAAX-1 (*RIT1*, SOS Ras/Rac (calcified aortic stenosis) guanine nucleotide-exchange factor (GEF)-1 (*SOS1*) genes), Turner (X0), Hennekam (ORPHA:2136, collagen and calcium-binding epidermal growth factor (EGF) domain-1 (*CCBE1*) gene) [8, 9].

## Diagnostic strategy

### Objectives

To confirm PIL diagnosis clinically, the differential diagnoses must be eliminated before proposing an adapted therapeutic strategy. The clinical history and symptomatology are similar for children and adults. Pediatric specificities will be noted, as necessary, in the text. Different healthcare professionals are involved in the initial diagnosis (Table 1).

**Table 1:** Healthcare professionals involved in the PIL initial diagnosis and therapeutic management

Specialist	Monitoring, screening for complications	Follow-up
Gastroenterologist, adult and pediatric, pediatrician, general practitioner	Growth, body mass index, edema, ascites	Child: every 3–6 months
Nutritionist	Nutritional status (fat-soluble vitamins, albumin)	Adult: annually
Internist	EFA deficiency in children	
	Complications	
	Compliance	
Dietitian	Energy intake	Child: every 3–6 months or even annually according to nutritional status and age
	MCT, EFA, fat-soluble vitamins and protein supplementation	Adult: every 1–3 years according to the nutritional status
Pneumologist, cardiologist	If complications are suspected: pericardial or pleural effusion	On request
Vascular medicine	Lymphedema management	On request
Thoracic, digestive surgeon	If complications are suspected: pericardial or pleural effusion	On request
Psychologist	Major impact on quality of life (strictly low-fat diet, lymphedema)	On request
Nurses	Patient education	Once, repeat as needed
Physiotherapist	Lymphedema treatment: low-stretch bandages, patient education	Reassessment at long term, if necessary
Podiatrist	Foot care if lower limb lymphedema	Every 6–12 months or more often if necessary
Orthotist	Supply elastic compression and bandaging materials for lymphedema	Every 3–6 months
Social worker	Recognition of disability, financial aid for products with little or no health-insurance coverage	On request
School doctor, child-protection agent	Specific program for inclusion at school	On request

*MCT* medium-chain triglyceride, *EFA* essential fatty acid

### Medical history

- Clinical history: age and circumstances of clinical sign onset (edema, serous or chylous effusions, diarrhea), the child’s other notable history
- Family history: consanguinity
- Other associated signs or malformations: lymphedema, yellow nail syndrome, systemic form with exudative enteropathy: Hennekam syndrome, Noonan syndrome,…

### Initial physical examination

- Edema

Peripheral edema, the principal clinical sign, reflects the decreased oncotic pressure resulting from hypoproteinemia. It varies in intensity, from moderate edema of the lower limbs, sometimes asymmetric, to more marked forms involving the face or external genitalia. Moderate serous effusions (pleural, pericardial, intraperitoneal) are frequent. However, effusions can be abundant and life-threatening in those forms (tamponade, compressive and asphyxiating pleural effusions) [1, 6]. PIL can be suspected during pregnancy by fetal ultrasound showing ascites and/or lower limb edema [10]. However, those signs have multiple causes and are not specific to PIL.

For very young children, when PIL is most often diagnosed, the edema does not yet affect the lower limbs because they are usually lying down and involves the face, lumbar region and male genitalia.

- Moderate and/or intermittent diarrhea is the main digestive symptom of PIL in children and young adults; however, it is non-specific and inconsistent [11, 12].
- Other clinical signs
  - Asthenia is a symptom frequently reported by patients, with notable fatigue, increased during viral infections (e.g., oropharyngeal).
  - Limb lymphedema is a rarer manifestation of PIL. It can be differentiated from hypoproteinemia-related edema: the cup sign in the infiltrated area is a less marked because of the lower fluid component than tissue component (skin thickening and fat deposition) of lymphedema. Lymphedema mainly affects the lower limbs below the knees (feet, ankles, calves) and more rarely the thighs; it can also affect the upper limbs (hand, forearm), thorax (breast), face and external genitalia (with thickening of the scrotal skin) [13, 14]. Clinically, it is not always easy to distinguish hypoproteinemia-related edema from lymphedema. The lymphedema diagnosis can be confirmed by the quasi-pathognomonic Stemmer’s sign (when the foot is involved): the inability to pinch the skin on the dorsal side or base of the 2nd toe demonstrates the presence of lymphedema-related skin thickening [15]. In adults, primary lower limb lymphedema may also be suggestive of pauci-symptomatic PIL [16].
  - An epigastric abdominal mass, attributed to a pseudomass created by parietal edematous infiltration of the small intestine, can be palpated [17].
  - Cutaneous pallor is sometimes caused by severe anemia (iron deficiency), and hypothetically would be attributed to chronic blood loss through possible small bowel ulcerations; however, externalized gastrointestinal bleeding remains a very rare and atypical feature of PIL [5, 18].
  - Subocclusion of the small intestine caused by either major localized parietal edema or more-or-less extensive pseudocystic dilations of the jejunal wall. It is rare and mimics tight jejunal stenosis. These lesions are identified after anatomopathological analysis of surgically excised specimens [19].
  - Chylous reflux into abdominal skin, lower extremities, perineum and external genitalia is suggestive of microcystic lymphangioma with numerous vesicles filled with a rarely described milky fluid [20].
  - Migratory necrolytic erythema [21].
  - Nail or digital clubbing results from a poorly understood mechanism [22].

### Particularities in children

PIL is usually diagnosed in children before the age of 3 years; some forms can be very extensive and lethal [6, 23–25]. The main clinical signs are, as in adults, edema, sometimes of the face and hands, associated or not with ascites or even pericardial effusion (chylous or not) of variable volume that can be life-threatening (tamponade). Frequently described non-specific digestive signs include diarrhea, abdominal pain, nausea or vomiting. Weight loss or absence of weight gain and growth retardation are common. Malabsorption can lead to symptomatic deficiency of fat-soluble vitamins (A, D, E and K), with episodes of hypocalcemia sometimes responsible for convulsions, tetany or osteomalacia [6, 25, 26]. An association with celiac disease has been reported [27].

## Syndromes associated with intestinal lymphangiectasias

Syndromes involving the lymphatic system—suggestive of overlapping syndromes among them—may include intestinal lymphangiectasias: yellow nail syndrome (also including pulmonary involvement with chronic cough, and frequently bronchiectasia, chronic sinusitis and lymphedema of the limbs), originally described in 1964 by Samman and White [28–31]; Hennekam syndrome linked to a mutation in the collagen and calcium binding epidermal growth factor (EGF) domain-1 *(CCBE1*) gene (mental retardation, seizures, lymphedema of the limbs and face; ORPHA: 2136) [32]; generalized lymphatic dysplasia linked to a mutation in the piezo-type mechanosensitive ion channel component-1 (*PIEZO1*) gene (ORPHA: 3202) [33]. Even more rare associations have been reported with tuberous sclerosis of Bourneville (tuberous sclerosis complex subunit-1, -2 genes (*TSC1*, *TSC2*; ORPHA: 805) [34], neurofibromatosis type-1 (von Recklinghausen disease, *NF1* gene; ORPHA: 636) [35] and CHAPLE syndrome (CD55 deficiency with hyperactivation of complement, angiopathic thrombosis and protein-losing enteropathy; ORPHA: 566175) [36]. WILD (warts, immunodeficiency, lymphedema and anogenital dysplasia; ORPHA: 568056) syndrome always includes multisegmental lymphedema present before 1 year and 70% of the cases are associated with PIL [37].

## Genetic counseling

To date, no gene has been implicated in PIL and familial forms are exceptional. Therefore, genetic counseling is not possible for isolated PIL but genetic analysis is useful in syndromic forms, such as Hennekam syndrome [32].

## Complementary examinations

The work-up is the same regardless of age PIL is suspected.

### Esophagogastroduodenoscopy (EGD) with duodenal biopsies

**Fig. 1:**
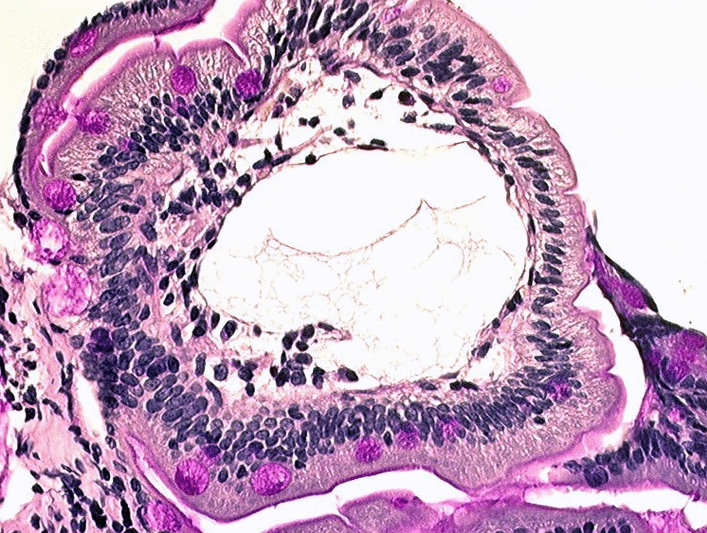
Lymphatic dilations in a duodenum biopsy (May-Grünwald Giemsa stain, ×40)

Intestinal lymphangiectasias can be visualized during EGD. Their macroscopic appearance is that of yellow/brown elevations (chylomicrons) of varying density and size. Systematic duodenal biopsies (even when the macroscopic aspect is normal) confirm the diagnosis with the presence of more-or-less voluminous and diffuse lymphatic dilations in the small intestine (Fig. 1). They contain a milky liquid consisting of pancreatic lipase-digested lipids and are located in the mucosa and submucosa (sometimes the serosa, which is not visible on biopsies), but have no cellular abnormalities. Sometimes, abnormalities may be minimal, with the mucosa simply edematous and non-creamy. In those situations, biopsies may be normal if they are taken in healthy areas, as lesions may be segmental, and thus do not exclude or confirm PIL diagnosis. To see lymphangiectasia, it is important that the patient follow a normal non-low-fat diet before EGD to obtain biopsies.

No villous atrophy or microorganism is seen in the digestive biopsies. It is important to retain that EGD and biopsies may be non-contributory if the involvement is segmental and/or after the duodenum. Thus, repeated endoscopies may be necessary to confirm the diagnosis with histological lymphangiectasia. A lipid load with ingestion of long-chain triglycerides (e.g., butter or olive oil) 24–72 h before EGD may increase its sensitivity [38].

### Capsule endoscopy

**Fig. 2:**
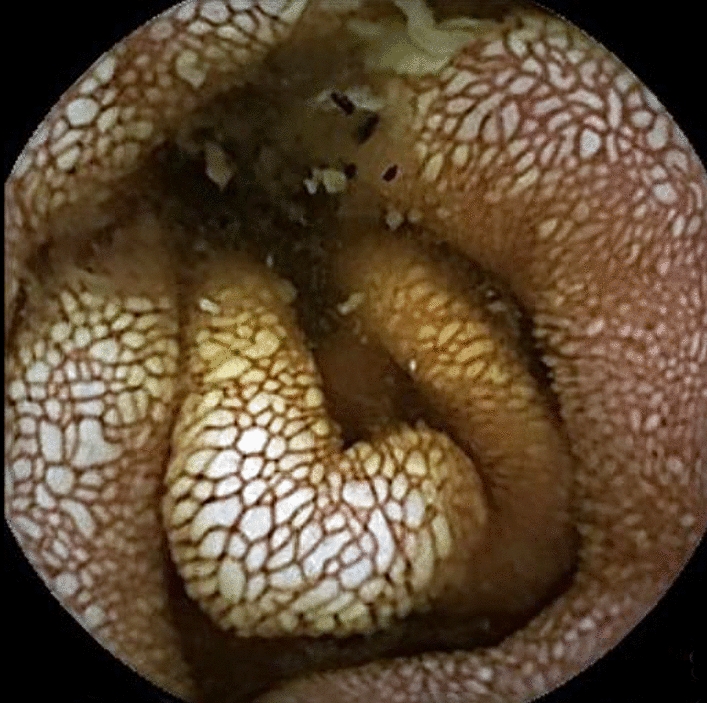
Capsule endoscopy: edematous aspect of the jejunum mucosa with whitish and swollen villi

This investigation enables severity, location (their preferential sites, i.e., proximal jejunum or distal ileum) and extent in the small intestine to be determined [39–41], but remains difficult to perform in infants. It enables PIL diagnosis if lesions are absent in the duodenum and therefore not seen during EGD. Lymphangiectasias are identified as whitish, millimetric, flat or raised spots, sometimes coalescing (Fig. 2). A lipid load (butter, olive oil) can also increase the sensitivity of capsule endoscopy [38]. Capsule endoscopy is a key examination for the initial diagnosis and also monitoring of lesion evolutions, especially in the case of clinical deterioration. In patients with severe anemia and iron deficiency, it can detect the rare presence of mucosal ulcerations, sometimes associated with lymphangiectasias. Finally, it can help identify intestinal lymphoma. Capsule endoscopy is also feasible in children but often requires that it be directly deposited in the stomach of those under 6–8 years of age during gastric endoscopy because of its difficult ingestion [42, 43]. It is useful for the initial diagnosis and also discussed to assess the extent of intestinal damage.

### Double balloon-enteroscopy/surgical biopsies

Enteroscopy is not considered a first-line procedure. It may be indicated to examine atypical areas (e.g., ulcerated) not accessible to conventional upper and lower endoscopies, to obtain biopsies to exclude differential diagnoses or complications, and, mainly, intestinal lymphoma [44]. If the targeted areas cannot be reached by enteroscopy and if an alternative diagnosis is suspected, a surgical intestinal biopsy may be warranted on a case-by-case basis.

Also on a case-by-case basis, double-balloon enteroscopy is another option in children over 6 years old, as it can obtain samples providing histological signs of PIL [45].

### Laboratory analyses

Indirect biological abnormalities are suggestive of PIL, which are the same for adults and children [25]. Hypoproteinemia, notably sometimes severe hypoalbuminemia (< 10 g/L), is constant. The absences of proteinuria and liver failure confirm the digestive origin of protein loss. The contrast between profound hypoalbuminemia and normal or moderately low prealbumin levels is characteristic of an exudative enteropathy. Hypogammaglobulinemia consists of low IgG, mainly IgG1, 2 and 4, while IgA and IgM levels remain subnormal [4]. Lymphopenia is frequent with a low CD4/CD8 ratio. Protein leakage into the digestive track related to exudative gastroenteropathy is confirmed by measurement of α_1_-antitrypsin clearance, which is significantly elevated (normal < 24 mL/24 h for children, < 15 mL/24 h in adults) [8]. α_1_-antitrypsin is a protein with anti-protease functions that is neither degraded nor absorbed in the intestine. In protein-losing enteropathy, all of the α_1_-antitrypsin seeping into the intestinal lumen remains intact in the stool and reflects the extent of exudation. This analysis requires blood sampling to determine the plasma α_1_-antitrypsin concentration and 24-h stool collection to measure total stool volume and α_1_-antitrypsin concentration [46].

Few data are available on specific deficiencies in patients with PIL but the risk is considered significant, particularly for EFAs and fat-soluble vitamins caused by malabsorption and prolonged strict low-fat diet, which can have deleterious consequences in the child and on his/her development [8, 47]. Zinc deficiency can be observed in the very rare cases with associated ulcerations that can also explain iron deficiency.

The status of vitamins, trace elements and EFAs must be assessed at diagnosis and regularly thereafter (at least once a year for adults and at least every 6 months for children). These analyses include dosages of fat-soluble vitamins (A, E and D), factors II, VII, IX and X for vitamin K, water-soluble vitamins (B9, B12, C) and trace elements (iron, copper, zinc, selenium), prothrombin time, and plasma and erythrocyte EFA chromatographies. When deficiency is documented, oral or even intravenous supplementation is essential.

### Other complementary examinations

**Fig. 3:**
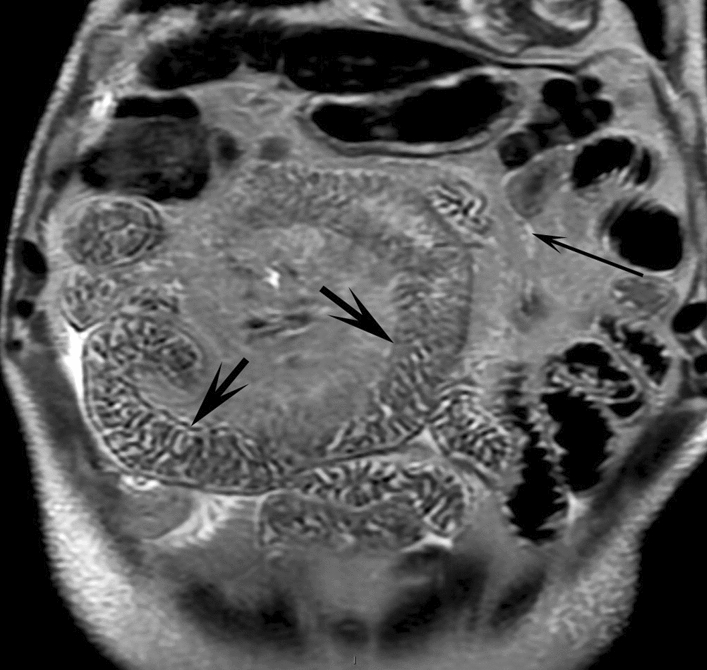
Non-contrast magnetic resonance (MR) lymphography. Note the parietal thickening of the small intestine, lymphangiectasias visible as hypersignals in small cavities (short arrows, and dilation of the lymphatic ductal network (long arrow)

- ^99m^Technetium–labeled human serum albumin scintigraphy is not widely used in France and has been replaced by α_1_-antitrypsin clearance. It shows intestinal hyperfixation indicating intraluminal protein loss. Sequential images obtained over 24 h are necessary to detect intermittent protein loss. Its sensitivity is very good and enables localization of the site of protein leakage [48].
- Abdominal ultrasonography is able to visualize indirect PIL signs in adults: dilation of a digestive loop, regular diffuse thickening of the intestinal wall, thickening of the folds, mesenteric edema and sometimes ascites [49]. It is rarely used for PIL diagnosis in children but can be useful to look for ascites.
- Abdominal and pelvic computed-tomography (CT) scan/enterography is the first exploration to eliminate abdominal compression (lymphatic, venous). Images are analyzed after contrast-medium injection. The images obtained are the same for adults and children: diffuse or sometimes nodular edema-induced parietal thickening of the small intestine wall. It is sometimes associated with digestive dilation forming a halo sign [50, 51]. CT scans can help confirm localized or segmental lesions. CT-enterography with ingestion of a product, e.g., oral mannitol, causing distension of the intestinal lumen may be useful to examine the bowel wall, to confirm a localized lesion of the small intestinal wall suggesting a complication or a differential diagnosis.
- Lymphoscintigraphy is the most useful exploratory investigation to analyze the lymphatic system of patients with lower and upper limb lymphedema. The tracer, ^99m^technetium-labeled albumin, is injected intradermally into the first interdigital space of the hands or feet. Because of its size, this colloid is captured only by the lymphatic capillaries. In PIL, lymphoscintigraphy is not usually used but it can show digestive tracer uptake in fewer than 50% of patients with histologically proven diagnoses [52]. Direct lymphography, using the iodinated contrast medium Lipiodol®, is no longer practiced in France, but is still used in other countries, coupled with CT scan. Its results can strongly suggest the diagnosis of PIL, with visualization of mesenteric edema, thickening of the intestinal wall, serous effusions (ascites, pericardium, pleural), digestive, lumbar, pleuropulmonary and/or mediastinal lymphatic reflux or absence of lymphatic flow in the thoracic duct [53].
- Non-contrast magnetic resonance (MR) lymphography is a technique without contrast-medium injection that exploits the very high signal of slow-moving liquids compared to that of solid organs. Thus, it is possible to analyze the lymphatic vessel network, lymph nodes and parietal anomalies, especially in the small intestine. In intestinal lymphangiectasia, MR imaging (MRI) usually shows parietal thickening that can involve the entire small intestine or predominate in the jejunum but also directly visualizes lymphatic dilations developed within the submucosa and the mucosa [54] (Fig. 3). It is not very easy to differentiate between lymphangiectasias of primary and secondary origin. The best argument supporting a PIL diagnosis is the association with other lymphatic abnormalities that can be seen in the retroperitoneum, spleen, thorax or limbs [55]. This imaging technique can be used in children (under sedation or general anesthesia, if necessary) to assess digestive involvement and sometimes more diffuse disease (pulmonary lymphangiectasias and/or lymphatic malformations, e.g., thoracic duct agenesis) [51, 56]. It can also visualize the lymphatic network when lower limb lymphedema is associated and allows lymphatic vessel classification as hypoplastic, aplastic or dysplastic [55].
- Immunological investigations are informative because of the B-and T-cell abnormalities found in patients with PIL. B-cell impairment is characterized by low immunoglobulin levels (mainly IgG, but also less affected IgA and IgM, in contrast to common variable immunodeficiency disorder (CVID) with intestinal lymphangiectasis and exudative enteropathy) and less Ig production after in vitro antigenic stimulation, perhaps attributable to a possible dysfunction of B- and T-cell collaboration [57]. B cell numbers are very moderately below normal (switched memory B cells). The abnormal distributions of T and B lymphocytes may mimic a late-onset combined immune-deficiency (LOCID) phenotype, with upper respiratory tract and pulmonary bacterial infections (hypogammaglobulinemia, fewer B lymphocytes, especially switched memory B lymphocytes), and opportunistic infections (fewer CD4^+^ T lymphocytes, especially naïve CD4^+^). However, PIL does not seem to expose these patients to more frequent opportunistic infections [58]. Deficient T-cell immunity is characterized by lymphopenia, prolonged rejection time after skin allografting and lower proliferative response in vitro to various stimulants (anti-CD3, anti-CD28). Furthermore, PIL patients’ peripheral blood has extremely low levels of CD4^+^ T cells, especially naive, CD45RA^+^CD62L^+^ or CD45RA^+^CCCR7^+^, while CD45RO^+^ memory cells are moderately below normal. Although CD45RA^+^CD45RO^+^CD8^+^ T cell numbers are also moderately below normal, they express elevated levels of lymphocyte-apoptosis markers [59].
- Cardiac ultrasonography and/or cardiac catheterization are required to rule out differential diagnoses in adults and children, especially a cardiac cause of exudative enteropathy. Transthoracic cardiac ultrasonography can be used to look for chronic constrictive pericarditis, which may also require cardiac catheterization to measure the pressures in the heart chambers [8, 46].

## Main differential diagnoses of exudative enteropathy and/or lymphangiectasias

Before confirming PIL diagnosis, it is necessary to exclude the other diagnoses responsible for lymphangiectasias associated with exudative gastroenteropathy or not [8, 46] (Table 2).

**Table 2:** Causes of exudative gastroenteropathies associated with lymphangiectasias or not, adapted from [8, 46]

Exudative gastroenteropathy	Causes
With lymphangiectasias^a^	Intestinal lymphoma
	Lymphoenteric fistula
	Whipple’s disease
	Crohn’s disease (lymphangiectasia inconstant)
	Sarcoidosis
	Sjögren’s syndrome
	Intestinal tuberculosis
	Systemic sclerosis
	Retroperitoneal fibrosis post-radio- and/or chemotherapy
	Human immunodeficiency virus-associated enteropathy
	Myeloma, Waldenström’s disease
	Amyloidosis
	Histiocytosis
	Venous hypertension downstream from the thoracic duct: constrictive pericarditis, venous thrombosis (superior vena cava, subclavian), Fontan procedure to treat cardiac malformations
Without lymphangiectasias	Menetrier's disease
	Systemic lupus erythematosus in adults and children
	Behçet's disease (possible telangiectasias)
	Common variable immune deficiency (CVID)

^a^Pathologists do not always look for and note the presence of lymphangiectasias for some pathologies

## Complications

### Lymphomas

Non-Hodgkin lymphomas developing during PIL, although rare, does not seem to be incidental. A recent literature review provided some details [60]. Since 1961, 14 “lymphomas” have been reported in adults followed for PIL. Only 11 were discussed because they are well-documented. Intestinal lymphangiectasia had been known for 3 to 45 years when lymphoma was diagnosed. Lymphomas were always of the B lineage, most often large cell (aggressive), and were in the digestive tract in 70% of the patients. Chemotherapy achieved disappearance of the exudative gastroenteropathy in 4/11 patients. Among the 11 analyzable patients, 9 were in remission and 2 died of complications. The mechanisms of lymphoma development in PIL are poorly documented. One of the hypotheses could be a possible B- or T-helper–cell immune deficiency, associated with a secondary deficiency with loss of immunoglobulins and lymphocytes in the digestive tract lymph [57].

### Infections

Although PIL patients have more-or-less severe hypogammaglobulinemia and lymphopenia, their risk of bacterial or opportunistic infections does not appear to be greater than in the general population or CVID patients with exudative enteropathy [4]. However, very rare hypogammaglobulinemia cases are associated with recurrent bacterial infections (sinusitis, *Klebsiella pneumoniae* lung disease, sometimes fatal for children) [61]. A single patient with group G streptococcal empyema and 3 others with disseminated cryptococcosis (meningitis, osteoarthritis) have been reported [62–64]. Disseminated and profuse human papillomavirus-related warts have been described in patients, most often—but not always—with associated lymphoma [65, 66]. Persistent diarrhea due to *Cryptosporidium sp.* was reported in 2 children with PIL, aged 1 and 12 years [67].

### Other complications

- Gelatinous transformation of the bone marrow is a very rare complication characterized by the replacement of hematopoietic cells and bone marrow adipocytes by an amorphous extracellular material composed of acidic mucopolysaccharides. One such case was reported in a patient with PIL and attributed to undernutrition secondary to malabsorption [68].
- Recurrent hemolytic uremic syndrome (HUS) associated with PIL and HUS with hypocomplementemia has been observed in several youths aged 17 and 23 years [69, 70]. It could have been caused by a CD45 deficiency unknown at the time of those publications.
- Hepatic fibrosis, a complication (or an associated entity) confirmed elastographically and histologically, is more-or-less reversible under a strict fat-free diet [71, 72].
- Malabsorption syndrome has been observed in elderly PIL patients and may be limited to osteomalacia caused by vitamin D deficiency [73].

## Therapeutic management and monitoring

Management has several objectives:

- To reduce the digestive loss of triglyceride-rich lymph or chyle by reducing long-chain–lipid intake, and increase albumin levels and thereby decrease edema;
- To provide fat-soluble vitamins (A, D, E and K) and avoid EFA deficiency;
- To replace long-chain fatty acids with medium-chain fatty acid (triglycerides) supplementation (6–12 carbon atoms) [74, 75];
- To evaluate the risk of possible complications, such as lymphoma;
- To examine the possible involvement of serous membranes with the risk of specific complications that may be life-threatening (pleural effusion, pericardial effusion with tamponade, ascites);
- In children, in addition to the above objectives, to avoid deficiencies and malnutrition (delayed growth).

### Strict low-fat diet enriched with medium-chain triglycerides (MCTs)

Initially seen as long term and sometimes definitive, this diet is the cornerstone of PIL management [74] (Table 3). However, in children—unlike adults—it is often possible to adopt a normal diet during disease evolution (Appendix 1). The strict low-fat diet impedes chylomicron formation, thereby lowering lymphatic pressure in the viscera, which in turn reduces lymphatic vessel dilation and chyle leakage. Lists of recommended and not recommended foods are mentioned in Appendix 2. MCT (including MCT oil) addition to the diet (i.e., at least 1 tablespoon of oil per meal for adults provides daily normal lipid intake) ensures lipid intake without absorption through the visceral lymphatic circulation because MCTs are not absorbed via the chylomicrons.

**Table 3:** Summary table of nutritional management

*Diet*	
Low-fat (< 10% total energy intake) MCT-enriched	
From birth: cow's milk protein-based infant formulas, enriched with MCTs and low in long-chain triglycerides	
As food intake is diversified, systematic addition of substitute fats throughout childhood (MCT oil)	
In adulthood: strictly low-fat diet (skim milk, lean meat and fish, 0% fat dairy products), addition of MCTs (MCT oil)	
*High-protein diet*	
For children: lean meat/fish, twice a day; sufficient consumption of 0% fat or lean dairy products (2 to 3 per day)	
For adults: lean meat/fish, 1 to 2 times a day; sufficient consumption of 0% fat or lean dairy products (2 per day minimum)	
*EFA intake by age*	
Infant milk/formula	
Addition of vegetable oils (e.g., rapeseed oil): at least 1 teaspoon, adapted to tolerance	
Consumption of oily fish (once a week)	
EFA capsules (adult or if child is able to swallow)	
*Oral fat-soluble vitamins*	
From birth: POLYVIT® ADEC 2 doses per day (0.6 mL per day) i.e., 6000 IU of vitamin A, 2000 IU of vitamin D, 100 mg of vitamin C, 10 mg of vitamin E	
Additional fat-soluble vitamin supplementation according to the dosages	
Vitamin A	
Vitamin D: 50,000 IU/ampoule for children (1 ampoule every 6 weeks); 100,000 IU/ampoule for adults	
Vitamin E: α-tocopherol, 17 mg/kg per day	
Vitamin K: 10 mg/mL, 1-mL vial if vitamin K deficiency causes hemostasis disorders	
*For the most severe forms*	
Intravenous lipid intake of emulsions such as 20% soybean, MCT, olive, fish oils, 2 g/kg/month IV with addition of water- and fat-soluble vitamins	

The use of specific dietary infant formulas (cow's milk protein-based formulas with more than 50% MCTs and EFAs) can cover the specific needs of this age group. They can be used for as long as possible, even after 3 years of age, to assure sufficient intake of EFAs, and other macronutrients and micronutrients difficult to achieve in the context of the low-fat diet (especially iron). As soon as the infant’s nutritional intake starts to be expanded, from about 4 months onward, long-chain lipid substitution with MCT oil should be started to provide MCTs. If the specific infant formula proves insufficient to cover EFA needs, daily intake of vegetable oil (alternating rapeseed oil and sunflower oil) in very small quantities may be recommended depending on clinical tolerance (either with classic vegetable oils or specific oils). For the most severely affected children, intravenous supplementation with EFA and fat-soluble vitamins may be prescribed.

In addition, a high-protein diet (up to 3 g/kg/day in adults) is essential to compensate for protein deficiency. If the patient’s dietary needs cannot be met, high-protein supplements without fat are useful. A gruel (thin)/mush (thick) containing only protein and carbohydrates can also assure major caloric and protein intake without incorporating fat for adults. The consumption of protein-rich dairy products without lipids, is another option for adults and older children.

After a few weeks on a fully compliant diet, clinical (edema) and biological (albumin, gammaglobulin, lymphocyte count) abnormalities tend to correct but not normalize. If the patient is regularly treated with albumin-infusions, their interval can be lengthened or the infusion discontinued [76]. Regular reevaluation of the low-fat diet is important if clinical inefficacy is observed. Should the strictly low-fat diet fail to achieve the desired effects, enteral nutrition and more rarely parenteral nutrition can be proposed [77].

Regular assessment—at least every 3 to 6 months—of the child’s diet is important. Improved clinical status could permit progressive aliment expansion under clinical and biological monitoring to meet children’s particularly high EFA needs, especially of omega 3, as they are precursors of docosahexaenoic acid [8, 47].

Regular monitoring of fat-soluble vitamins and EFAs (chromatography) is essential to detect possible deficiencies that can appear on a strictly low-fat diet [78]. Intake of vitamins A, D, E and K must be ensured for all children (POLYVIT® ADEC, 2 doses per day or even with the addition of vitamins depending on the dosage). Should oral intake be insufficient, intravenous supplementation may be indicated (20% lipid emulsion 2 g/kg/month with the addition of water- and fat-soluble vitamins). Drugs with different names may be put on the market or the content of the existing ones may be changed. Recommended vitamin dosages are noted in Table 3.

### Enteral and parenteral nutrition

Patients not responding to a strictly low-fat diet suffer severe undernutrition. Enteral nutrition with an elemental, semi-elemental or polymeric product but still low fat may be necessary. No differences among the efficacies of the different types of enteral nutrition have been demonstrated [77]. Measurements of vitamin and trace-element concentrations in blood is recommended to determine whether IV supplementation is necessary. Measurement and regular IV infusion of EFAs is also required in addition to enteral nutrition. In very rare cases, parenteral nutrition may be required [77]. Very young infants require a strict low-fat diet without oils, and regular (monthly or bimonthly) IV lipid and vitamin infusions.

### Octreotide

In 1998, Ballinger and Farthing proposed treating PIL with octreotide, a somatostatin analogue [79]. Since then, octreotide has been regularly prescribed for PIL, whether isolated or associated with Hennekam syndrome. The octreotide dose ranges from 1 to 10 µg/kg, given in two subcutaneous injections per day for adults, and 15 to 20 µg/kg, twice daily, for children, and if clinical tolerance is good, relayed by its slow-release formulation, 20–30 mg every 4 weeks for adults [80, 81]. Octreotide is always used in combination with the low-fat diet. Although no prospective randomized study on octreotide efficacy has been reported, the drug has been reported to be associated with inconsistent and variable improvement of clinical, biological (increased albumin) and histological abnormalities in adults and children [80–82]. Its mechanisms of action on the gut remain unknown and may include: reduced acetylcholine release in intestinal plexuses (effects on absorption and motility), decreased fat and triglyceride absorptions in the thoracic duct, diminished intestinal blood flow and release of vasoactive peptides linked to local microscopic inflammation [82].

### Sirolimus/everolimus

Sirolimus, a mammalian target of rapamycin (mTOR)-receptor inhibitor, is used to treat vascular malformations with a lymphatic component [83, 84]. Sirolimus has anti-lymphangiogenic properties that impair vascular endothelial growth factor-A (VEGFA) and -C (VEGFC) signaling in lymphatic endothelial cells responsible for the action in PIL. Moreover, the report of PIL associated with tuberous sclerosis of Bourneville with *TSC2*-gene mutation in a 2-year-old child described a favorable evolution with sirolimus progressively increased to 0.61 mg/kg and plasma levels reaching 10.7 ng/mL (reference level: 3.0–18.0 ng/mL) [34]. The initial sirolimus dose for adults is 2 mg/day, with 2 blood determinations per week at baseline and a target plasma level of 5–15 ng/mL [11, 85, 86]. For children, by analogy with vascular malformations, sirolimus is started at 0.08–0.1 mg/kg/day, ideally in 2 doses, targeting the same residual level goal as in adults [87]. For adults and children, the tests required for sirolimus prescription are the same:

- Before prescription: blood count, blood ionogram, urea, creatininemia, liver function tests, cholesterol, triglycerides, glycemia; human immunodeficiency virus, hepatitis B, C and β-human chorionic gonadotrophin (for women of childbearing age) serologies, tuberculin test/Quantiferon®, chest X-ray if risk of tuberculosis, stool parasitology to look for anguillulosis and, if a risk factor, preventive ivermectin;
- During treatment monitoring: serum residual sirolimus concentration every 15 days to reach the therapeutic range (4–15 ng/mL), then once a month, blood count, ionogram, urea, creatininemia, liver function tests, cholesterol, triglycerides, glycemia.

Everolimus, another mTOR inhibitor, was given to a 12-year-old starting at 1.6 mg/m^2^ per day and then adapted to obtain a plasma level of 5–15 ng/mL; it achieved a partial response: diarrhea disappeared, gamma globulins increased, albumin-infusion intervals could be prolonged [88]. mTOR-receptor inhibitor allows for personalized treatment focused on lesion extension and PIL severity but requires further study to confirm its efficacy.

**Fig. 4:**
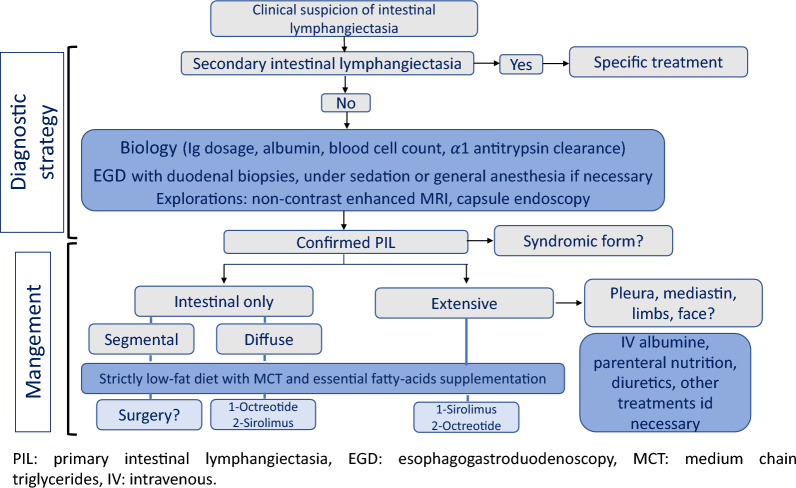
Management strategy for children with PIL adapted from [86]

For children, the following management strategy is proposed:

1. Secondary causes, the same as for adults, are rare but must be systematically excluded;
2. Extent of digestive involvement should be determined by imaging (non-contrast MR lymphography or endoscopic videocapsule, if possible);
3. In all cases, the main objective is to control the exudative enteropathy by a strict low-fat, high-protein diet or, if necessary, strict digestive rest with exclusive parenteral nutrition [8];
4. For the rare localized digestive forms, surgical treatment (resection and/or embolization) may be considered. For diffuse digestive forms, drug treatments, such as octreotide and sirolimus, may be indicated;
5. For PIL associated with serous effusion, sirolimus may be indicated before octreotide because of its anti-vascular action. This approach adapted to the PIL extent of was recently proposed by Kwon et al. after a retrospective study of 7 cases and a review of the literature [86] (Fig. 4).

### Other treatments

- Albumin infusions, diuretics, immunoglobulins, anticoagulants, vitamin therapy
  - Albumin infusions are a useful symptomatic treatment for large serous effusions or voluminous and extensive lower limb edema. They are administered at variable frequency (every 1–3 weeks), with the dose based on clinical (edema, effusions) and/or biological objectives. Their effect is very transient due to the persistent digestive protein loss. For children, it is usual to infuse 1 g/kg of 20% albumin over 2–3 h followed by injection of a diuretic, such as furosemide at 0.5–1 mg/kg.
  - Diuretics (furosemide, spironolactone) may sometimes be necessary to address persistent and disabling edema in adults.
  - Subcutaneous Ig injections are sometimes given, possibly combined with octreotide, if the patient experiences repeated bacterial infections attributed to profound hypogammaglobulinemia [61]. Ig supplementation may be necessary because of the digestive leakage, making reevaluation of the benefit/risk balance essential. Normal or subnormal IgA levels could hypothetically contribute to limiting the risk of infections. For children, Ig supplementation may be an option to treat profound hypogammaglobulinemia even in the absence of recurrent infections.
  - The indications for anticoagulants are the same as those for the general population, as the thrombotic risk is not increased despite frequent and sometimes severe hypoalbuminemia (< 15 g/L).
  - Fat-soluble vitamins supplementation is required for very young children (POLYVIT® ADEC, 1 or even 2 doses dose per day). For older children, vitamin A (A 313) and/or vitamin E will be necessary depending on their vitamin dosages. The current recommendations for vitamin D are 50,000 IU every 6 weeks and must be adapted according to regular level determinations. For patients requiring IV lipid intake (lipid emulsions), fat-soluble vitamins are provided by different vitamin mixtures.
  - Although bleeding is not characteristic or exceptional in PIL, it is sometimes useful to treat iron deficiency (orally or parenterally).
- Although other treatments have been tried, after or associated with strictly low-fat diet and MCT supplementation, their efficacies have not been evaluated by studies with rigorous methodology. For children, especially the youngest (before 12 months), propranolol has been prescribed in analogy with its use to treat hemangiomas but no large-scale trial has been undertaken [89, 90].
- Surgery
  - Digestive surgery has very limited indications in PIL treatment. However, when lymphangiectasias are segmental and very localized, their resection achieves major improvement of clinical and biological parameters. Thus, limited intestinal resections have also been performed in children with segmental forms of PIL or on a large macroscopic lesion [86, 91, 92]. Recently, lymphatic embolization (which can be repeated several times) has demonstrated efficacy for territory-limited PIL in a few children and may be considered a first-line therapy [93].
  - Thoracic surgery can be contributory for recurrent or large pleural effusions that may be life-threatening. When they are bilateral, the effusions are generally the consequence of hypoproteinemia and hypoalbuminemia leading to fluid leakage from the lymphatic and systemic vessels that have become more permeable towards the interstitium and the pleural and pericardial cavities. Damaged lymphatic vessel walls secondary to recurrent pleural effusions has been suggested in patients with associated yellow nail syndrome [94]. These effusions may or may not be chylous. When the effusion is large, it should be drained with a small caliber drain (about 16 Fr). This complete evacuation allows the pleural cavity (space between the parietal pleura and the visceral pleura, the real “skin” of the lung) to regain its shape and its almost “virtual” volume. Should that space and the fluid leakage persist, pleurodesis is an option to facilitate the symphysis between parietal and visceral pleura. Talc pleurodesis is undertaken to treat disabling malignant effusion and, according to an analysis of 4 very homogeneous trials, achieved effective symphysis in more than 80% of cases if lung reexpansion was obtained [95]. It is the simplest and most effective technique for PIL, and is usually done under videothoracoscopy, which assures visual control. For patients in poor general condition, talc pleurodesis can be done under local anesthesia, either directly by instillation through the drain or under videothoracoscopy. Indeed, talc pleurodesis under videothoracoscopy was considered significantly superior to that obtained by injection of the suspension through the drain in the meta-analysis by Dipper et al. [96]. However, based on their meta-analysis, Mummadi et al. considered the 2 techniques to be equivalent. Parietal pleurectomy, sometimes done to resolve recurrent pleural effusions, should not be considered a first-line procedure if a malformation of the lymphatic vessels in the parietal pleura is suspected. Indeed, pleurectomy can appear radical to obtain symphysis, as it alters the parietal lymphatics, which can then increase the leakage of lymph or even chyle. Thus, in PIL, talc pleurodesis should be the first-line therapy-of-choice to obtain pleural symphysis.
- Lymphedema treatment

French recommendations for primary lymphedema management were published in 2021 [97]. Schematically, treatment includes a volume-reduction phase, based on low-stretch bandages, followed by a maintenance phase, based on wearing elastic compression garments. It includes skin care to prevent cellulitis [98], physical exercise, and eventually manual lymphatic drainage, which has no effect on volume reduction [97] (Table 4). Bandaging is recommended as early as possible to avoid skin thickening and adipose tissue deposition, and should be taught to parents during therapeutic education sessions with a physiotherapist. Custom-made compression garments with the highest tolerated pressure can be worn as soon as the child is able to walk. Video tutorials made with physiotherapists are available for parents to learn how to use the bandages.

**Table 4:** Two phases of decongestive physiotherapy to treat lymphedema [97]

Phase 1: intensive phase (volume reduction)	Phase 2: maintenance phase (maintain decreased volume)
Low-stretch bandages 24 h/24: 5 days to 3 weeks according to the volume	Elastic compression during the day (every day, from morning to evening)
Exercises with bandages in place	(Self-applied) low-stretch bandages (3 nights/week)
Manual lymph drainage	Exercises
	Skin care
Skin care	Manual lymph drainage if necessary

## Follow-up—evolution

Life-long follow-up is necessary and globally similar for adults and children and involved some professionals (Table 1). It can be done in a consultation or in a day hospital at an adapted frequency (at least once a year for adults, every 3–6 months for young children). This multimodal reassessment is essential for all patients, whether or not they are receiving enteral or parenteral nutrition:

- Physical examination and nutritional status: edema assessment, weight, look for complication (infectious, neoplastic);
- Evaluation and consultation with the dietician to assure the strict adherence to the dietary restrictions and the intake of nutritional supplements (EFAs, MCTs, fat-soluble vitamins). with repletion of the dietary recommendations;
- Standard laboratory tests (albumin level, blood count, ionogram), vitamin dosages, trace elements and EFA (plasma EFA and erythrocyte membrane chromatographies).

Look for complications guided by questioning (weight loss, fever, asthenia…) and physical examination (auscultation, abdominal palpation, lymph node areas…) or even complementary investigations (ultrasound, scanner…) if lymphoma, serous effusion… suspected. Adaptation of lymphedema treatment according to volume and the patient's objectives (low-stretch bandages, elastic compression garments).

## Transition from pediatric to adult care

Change of practitioner should be discussed as of 15 years of age and be completed by 20 years; consultations can alternate between pediatric and adult services, or even consultations with referents from both services. This consultation allows reevaluation of the potential PIL-induced damage and complications, and its treatment. Maintaining the low-fat diet, and compliance to vitamin therapy and MCT supplementation should be addressed. The transition process from pediatric to adult care begins in early adolescence and should lead to the patient's appropriation of the disease and its required care. Part of the support for the adolescent is his or her empowerment process, and must respond to his or her concerns and questions, along with appropriate lifestyle adaptations. It is necessary to encourage and buttress the adolescent's understanding of PIL, using tools such as shared medical decision-making and specific education workshops, without the parents, to facilitate the transfer to personal autonomy.

## Patient education

Life-long patient education is essential to explain the pathology, especially to assure optimal follow-up of the strict low-fat diet. Education includes individual meetings with the dietitian and also with group workshops, particularly during adolescence to encourage the transition to the self-empowerment. The aims of patient education are to acquire knowledge about PIL, treatments and dietary recommendations. Workshops include the acquisition of management competence (e.g., percentage of fat in various common foods) and technical advice (recipes for using MCT oils). The social impact cannot be minimized because managing the low-fat diet is difficult in community settings, especially for the young. Psychological support of the child/adolescent may be necessary to overcome the difficulties encountered with the strict fat-free diet.

## Pregnancy

Little is known about the impact of PIL on the woman and her fetus. A few case reports have described normal pregnancies with no consequences for the newborns if adherence to dietary guidelines and treatment is maintained [99].

## Role of patient-support groups

No patient-support groups exist at present for pediatric or adult PIL. However, since many patients also have lymphedema of the limbs, PIL patients can participate in some patient-support groups for lymphedema.

Patient-support groups have several roles:

- To provide information to patients and their relatives in printed documents and information reviews, by organizing information meetings, with the participation of professionals, throughout France, thereby participating in their education, raising public awareness and disseminating information to non-specialist professionals on PIL;
- To organize therapeutic education-program workshops, day sessions or short-term stays promoting lymphedema self-management (self-massage and self-monitoring) coordinated with professional caregivers;
- To create meeting spaces and telephone hotlines, facilitating exchanges of experiences;
- To contact or initiate interactions with public authorities to improve patient management and quality of life;
- To represent the patients in the various health-dedicated institutions;
- To stimulate and contribute to financing for research on PIL and its treatment.

## Conclusion

PIL is a chronic disease requiring a prolonged and restrictive strict low-fat diet supplemented with MCTs and fat-soluble vitamins. The patient’s quality of life is often impaired by edema and/or lymphedema and the constraining diet. PIL’s evolution can be complicated by serous effusions (pleural, pericardial) with a more-or-less notable functional impact with the most severe forms potentially life-threatening. Non-Hodgkin lymphoma is a rare complication. Long-term multidisciplinary clinical and biological monitoring is necessary.

## Supplementary Information

Additional file1 (DOCX 19 kb)

## Data Availability

Not applicable.

## References

[1] Waldmann TA, Steinfeld JL, Dutcher TF, Davidson JD, Gordon RS. The role of the gastrointestinal system in “idiopathic hypoproteinemia.” Gastroenterology. 1961;41:197–207. 13782654

[2] Hokari R, Kitagawa N, Watanabe C, Komoto S, Kurihara C, Okada Y, et al. Changes in regulatory molecules for lymphangiogenesis in intestinal lymphangiectasia with enteric protein loss. J Gastroenterol Hepatol. 2008;23(7Pt2):e88-95. 18005011 10.1111/j.1440-1746.2007.05225.x

[3] Finegold DN, Schacht V, Kimak MA, Lawrence EC, Foeldi E, Karlsson JM, et al. *HGF* and *MET* mutations in primary and secondary lymphedema. Lymphat Res Biol. 2008;6:65–8. 18564920 10.1089/lrb.2008.1524 PMC4298750

[4] Sanges S, Germain N, Vignes S, Séguy D, Stabler S, Etienne N, et al. Protein-losing enteropathy as a complication and/or differential diagnosis of common variable immunodeficiency. J Clin Immunol. 2022;42:1461–72. 35737255 10.1007/s10875-022-01299-1

[5] Vignes S, Bellanger J. Primary intestinal lymphangiectasia (Waldmann’s disease). Orphanet J Rare Dis. 2008;3:5. 18294365 10.1186/1750-1172-3-5 PMC2288596

[6] Vardy PA, Lebenthal E, Shwachmann H. Intestinal lymphangiectasia: a reappraisal. Pediatrics. 1975;55:842–50. 1134884

[7] Le Bougeant P, Delbrel X, Grenouillet M, Leou S, Djossou F, Beylot J, et al. Maladie de Waldmann familiale. Ann Med Interne. 2000;151:511–2. 11104932

[8] Braamskamp MJ, Dolman KM, Tabbers MM. Clinical practice. Protein-losing enteropathy in children. Eur J Pediatr. 2010;169:1179–85. 20571826 10.1007/s00431-010-1235-2 PMC2926439

[9] Wang N, Shi W, Jiao Y. A *PTPN11* mutation in a woman with Noonan syndrome and protein-losing enteropathy. BMC Gastroenterol. 2020;20:34. 32054441 10.1186/s12876-020-01187-1 PMC7017519

[10] Schmider A, Henrich W, Reles A, Vogel M, Dudenhausen JW. Isolated fetal ascites caused by primary lymphangiectasia: a case report. Am J Obstet Gynecol. 2001;184:227–8. 11174507 10.1067/mob.2001.106756

[11] Na JE, Kim JE, Park S, Kim ER, Hong SN, Kim YH, Chang DK. Experience of primary intestinal lymphangiectasia in adults: twelve case series from a tertiary referral hospital. World J Clin Cases. 2024;12:746–57. 38322684 10.12998/wjcc.v12.i4.746 PMC10841145

[12] Lee WS, Boey CC. Chronic diarrhoea in infants and young children: causes, clinical features and outcome. J Paediatr Child Health. 1999;35:260–3. 10404446 10.1046/j.1440-1754.1999.00356.x

[13] Goktan C, Pekindil G, Orguc S, Coskun T, Serter S. Bilateral breast edema in intestinal lymphangiectasia [letter]. Breast J. 2005;11:360. 16174162 10.1111/j.1075-122X.2005.21578.x

[14] Wang X, Jin H, Wu W. Primary intestinal lymphangiectasia manifested as unusual edemas and effusions: a case report. Medicine. 2016;95: e2849. 26962779 10.1097/MD.0000000000002849 PMC4998860

[15] Vignes S. Les lymphœdèmes: du diagnostic au traitement. Rev Med Interne. 2017;38:97–105. 27591818 10.1016/j.revmed.2016.07.005

[16] Boursier V, Vignes S. Lymphangiectasies intestinales primitives (maladie de Waldmann) révélées par un lymphœdème des membres. J Mal Vasc. 2004;29:103–6. 15229406 10.1016/s0398-0499(04)96722-4

[17] Rao R, Shashidhar H. Intestinal lymphangiectasia presenting as abdominal mass. Gastrointest Endosc. 2007;65:522–3 (**discussion 523**). 17321261 10.1016/j.gie.2006.10.026

[18] Maamer AB, Baazaoui J, Zaafouri H, Soualah W, Cherif A. Primary intestinal lymphangiectasia or Waldmann’s disease: a rare cause of lower gastrointestinal bleeding. Arab J Gastroenterol. 2012;13:97–8. 22980601 10.1016/j.ajg.2012.03.001

[19] Lenzhofer R, Lindner M, Moser A, Berger J, Schuschnigg C, Thurner J. Acute jejunal ileus in intestinal lymphangiectasia. Clin Investig. 1993;71:568–71. 8374252 10.1007/BF00208483

[20] O’Driscoll JB, Chalmers RJ, Warnes TW. Chylous reflux into abdominal skin simulating lymphangioma circumscriptum in a patient with primary intestinal lymphangiectasia. Clin Exp Dermatol. 1991;16:124–6. 2032374 10.1111/j.1365-2230.1991.tb00322.x

[21] Baricault S, Soubrane JC, Courville P, Young P, Joly P. Erythème nécrolytique migrateur au cours d’une maladie de Waldmann. Ann Dermatol Venereol. 2006;133:693–6. 17053741 10.1016/s0151-9638(06)70994-2

[22] Wiedermann CJ, Kob M, Benvenuti S, Carella R, Lucchin L, Piazzi L, et al. Digital clubbing in primary intestinal lymphangiectasia: a case report. Wien Med Wochenschr. 2010;160:431–6. 20812055 10.1007/s10354-010-0815-0

[23] Munck A, Sosa Valencia G, Faure C, Besnard M, Ferkdadji L, Cézard JP, et al. Suivi de long cours des lymphangiectasies intestinales primitives de l’enfant. A propos de six cas. Arch Pediatr. 2002;9:388–91. 11998426 10.1016/s0929-693x(01)00799-0

[24] Wen J, Tang Q, Wu J, Wang Y, Cai W. Primary intestinal lymphangiectasia: four case reports and a review of the literature. Dig Dis Sci. 2010;55:3466–72. 20198428 10.1007/s10620-010-1161-1

[25] Kwon Y, Kim MJ. The update of treatment for primary intestinal lymphangiectasia. Pediatr Gastroenterol Hepatol Nutr. 2021;24:413–22. 34557394 10.5223/pghn.2021.24.5.413 PMC8443852

[26] Thapaliya I, Yadav J. Hypocalcaemic tetany linked to vitamin D deficiency and hypomagnesemia in primary intestinal lymphangiectasia: a literature review. Ann Med Surg. 2024;86:2049–57. 10.1097/MS9.0000000000001850 PMC10990415 38576918

[27] Perisic VN, Kokai G. Coeliac disease and lymphangiectasia. Arch Dis Child. 1992;67:13–6. 10.1136/adc.67.1.134 PMC1793534 1739329

[28] Samman P, White WF. The “yellow nail” syndrome. Br J Dermatol. 1964;76:153–7. 14140738 10.1111/j.1365-2133.1964.tb14499.x

[29] Vignes S, Baran R. Yellow nail syndrome: a review. Orphanet J Rare Dis. 2017;12:42. 28241848 10.1186/s13023-017-0594-4 PMC5327582

[30] Desramé J, Béchade D, Patte JH, Jean R, Karsenti D, Coutant G, et al. Syndrome des ongles jaunes associé à des lymphangiectasies intestinales. Gastroenterol Clin Biol. 2000;24:837–40. 11011259

[31] Benassaia E, Abba S, Fourgeaud C, Mihoubi A, Vignes S. Yellow nail syndrome: analysis of 23 consecutive patients and effect of combined fluconazole-vitamin-E treatment. Dermatology. 2024;240:343–51. 38071959 10.1159/000535577

[32] Hennekam RC, Geerdink RA, Hamel BC, Hennekam FA, Kraus P, Rammeloo JA, et al. Autosomal recessive intestinal lymphangiectasia and lymphedema, with facial anomalies and mental retardation. Am J Med Genet. 1989;34:593–600. 2624276 10.1002/ajmg.1320340429

[33] Fotiou E, Martin-Almedina S, Simpson MA, Lin S, Gordon K, Brice G, et al. Novel mutations in *PIEZO1* cause an autosomal recessive generalized lymphatic dysplasia with non-immune hydrops fetalis. Nat Commun. 2015;6:8085. 26333996 10.1038/ncomms9085 PMC4568316

[34] Pollack SF, Geffrey AL, Thiele EA, Shah U. Primary intestinal lymphangiectasia treated with rapamycin in a child with tuberous sclerosis complex (TSC). Am J Med Genet A. 2015;167A:2209–12. 25943403 10.1002/ajmg.a.37148

[35] Calabrese C, Pironi L, Di Febo G. Capsule endoscopy revealing small-intestinal lymphangiectasia and GI stromal tumor polyps in neurofibromatosis type 1. Gastrointest Endosc. 2006;64:130–1. 16813823 10.1016/j.gie.2006.01.032

[36] Ozen A, Comrie WA, Ardy RC, Domínguez Conde C, Dalgic B, Beser ÖF, et al. CD55 deficiency, early-onset protein-losing enteropathy, and thrombosis. N Engl J Med. 2017;377:52–61. 28657829 10.1056/NEJMoa1615887 PMC6690356

[37] Mansour S, Josephs KS, Ostergaard P, Gordon K, van Zanten M, Pearce J, et al. Redefining WILD syndrome: a primary lymphatic dysplasia with congenital multisegmental lymphoedema, cutaneous lymphovascular malformation, CD4 lymphopaenia and warts. J Med Genet. 2023;60(1):84–90. 34916230 10.1136/jmedgenet-2021-107820 PMC9811088

[38] Lee J, Kong MS. Primary intestinal lymphangiectasia diagnosed by endoscopy following the intake of a high-fat meal. Eur J Pediatr. 2008;167:237–9. 17453239 10.1007/s00431-007-0445-8

[39] Chamouard P, Nehme-Schuster H, Simler JM, Finck G, Baumann R, Pasquali JL. Videocapsule endoscopy is useful for the diagnosis of intestinal lymphangiectasia. Dig Liver Dis. 2006;38:699–703. 16527553 10.1016/j.dld.2006.01.027

[40] Vignes S, Bellanger J. Intérêt de l’entéroscopie par vidéocapsule dans le diagnostic des lymphangiectasies intestinales primitives. Rev Med Interne. 2007;28:173–5. 17229491 10.1016/j.revmed.2006.11.019

[41] Safatle-Ribeiro AV, Iriya K, Couto DS, Kawaguti FS, Retes F, Ribeiro U Jr, et al. Secondary lymphangiectasia of the small bowel: utility of double balloon enteroscopy for diagnosis and management. Dig Dis. 2008;26:383–6. 19188732 10.1159/000177027

[42] Rivet C, Lapalus MG, Dumortier J, Le Gall C, Budin C, Bouvier R, et al. Use of capsule endoscopy in children with primary intestinal lymphangiectasia. Gastrointest Endosc. 2006;64:649–50. 16996365 10.1016/j.gie.2006.03.008

[43] van der Reijden SM, van Wijk MP, Jacobs MA, de Meij TG. Video capsule endoscopy to diagnose primary intestinal lymphangiectasia in a 14-month-old child. J Pediatr Gastroenterol Nutr. 2017;64: e161. 28333772 10.1097/MPG.0000000000001586

[44] Lai Y, Yu T, Qiao XY, Zhao LN, Chen QK. Primary intestinal lymphangiectasia diagnosed by double-balloon enteroscopy and treated by medium-chain triglycerides: a case report. J Med Case Rep. 2013;7:19. 23316917 10.1186/1752-1947-7-19 PMC3565923

[45] Lin TK, Erdman SH. Double-balloon enteroscopy: pediatric experience. J Pediatr Gastroenterol Nutr. 2010;51:429–32. 20531023 10.1097/MPG.0b013e3181d2979c

[46] Amiot A. Gastro-entéropathies exsudatives. Rev Med Interne. 2015;36:467–73. 25618488 10.1016/j.revmed.2014.12.001

[47] Martinat M, Rossitto M, Di Miceli M, Layé S. Perinatal dietary polyunsaturated fatty acids in brain development, role in neurodevelopmental disorders. Nutrients. 2021;13:1185. 33918517 10.3390/nu13041185 PMC8065891

[48] Chiu NT, Lee BF, Hwang SJ, Chang JM, Liu GC, Yu HS. Protein-losing enteropathy: diagnosis with ^99m^Tc-labeled human serum albumin scintigraphy. Radiology. 2001;219:86–90. 11274540 10.1148/radiology.219.1.r01ap2986

[49] Maconi G, Molteni P, Manzionna G, Parente F, Bianchi PG. Ultrasonographic features of long-standing primary intestinal lymphangiectasia. Eur J Ultrasound. 1998;7:195–8. 9700215 10.1016/s0929-8266(98)00037-8

[50] Fakhri A, Fishman EK, Jones B, Kuhadja F, Siegelman SS. Primary intestinal lymphangiectasia: clinical and CT findings. J Comput Assist Tomogr. 1985;9:767–70. 4019833

[51] Malone LJ, Fenton LZ, Weinman JP, Anagnost MR, Browne LP. Pediatric lymphangiectasia: an imaging spectrum. Pediatr Radiol. 2015;45:562–9. 25301383 10.1007/s00247-014-3191-x

[52] So Y, Chung JK, Seo JK, Ko JS, Kim JY, Lee DS, et al. Different patterns of lymphoscintigraphic findings in patients with intestinal lymphangiectasia. Nucl Med Commun. 2001;22:1249–54. 11606892 10.1097/00006231-200111000-00013

[53] Sun X, Shen W, Chen X, Wen T, Duan Y, Wang R. Primary intestinal lymphangiectasia: multiple detector computed tomography findings after direct lymphangiography. J Med Imaging Radiat Oncol. 2017;61:607–13. 28345300 10.1111/1754-9485.12606

[54] Liu NF, Lu Q, Wang CG, Zhou JG. Magnetic resonance imaging as a new method to diagnose protein losing enteropathy. Lymphology. 2008;41:111–5. 19013878

[55] Arrivé L, Monnier-Cholley L, Cazzagon N, Wendum D, Chambenois E, El Mouhadi S. Non-contrast MR lymphography of the lymphatic system of the liver. Eur Radiol. 2019;29:5879–88. 30937582 10.1007/s00330-019-06151-6

[56] Brownell JN, Biko DM, Mamula P, Krishnamurthy G, Escobar F, Srinivasan A, et al. Dynamic contrast magnetic resonance lymphangiography localizes lymphatic leak to the duodenum in protein-losing enteropathy. J Pediatr Gastroenterol Nutr. 2022;74:38–45. 34406998 10.1097/MPG.0000000000003287 PMC8714618

[57] Heresbach D, Raoul JL, Genetet N, Noret P, Siproudhis L, Ramee MP, et al. Immunological study in primary intestinal lymphangiectasia. Digestion. 1994;55:59–64. 7509299 10.1159/000201124

[58] Malphettes M, Gérard L, Carmagnat M, Mouillot G, Vince N, Boutboul D, et al. DEFI Study Group. Late-onset combined immune deficiency: a subset of common variable immunodeficiency with severe T cell defect. Clin Infect Dis. 2009;49:1329–38. 19807277 10.1086/606059

[59] Vignes S, Carcelain G. Increased surface receptor Fas (CD95) levels on CD4^+^ lymphocytes in patients with primary intestinal lymphangiectasia. Scand J Gastroenterol. 2009;44:252–6. 18855225 10.1080/00365520802321220

[60] Hu D, Cui X, Ren W, Zhang J, Guan X, Jiang X. A case of primary intestinal lymphangiectasia with non-Hodgkin lymphoma. BMC Gastroenterol. 2021;21:461. 34895151 10.1186/s12876-021-01997-x PMC8665534

[61] Chegini S, Hershey PA. Successful management of primary intestinal lymphangiectasia with subcutaneous immunoglobulin (SCIG) and octreotide. Clin Immunol. 2010;135:319–20.

[62] Cole SL, Ledford DK, Lockey RF, Daas A, Kooper J. Primary gastrointestinal lymphangiectasia presenting as cryptococcal meningitis. Ann Allergy Asthma Immunol. 2007;98:490–2. 17521035 10.1016/S1081-1206(10)60765-X

[63] Mathurin M, Devatine S, Kopp-Derouet A, Guillonnet A, Alanio A, Lourenco N, et al. Cryptococcal meningitis and cerebral vasculitis in a patient with primary intestinal lymphangiectasia: a case report. Eur J Clin Microbiol Infect Dis. 2023;42:1263–7. 37668805 10.1007/s10096-023-04657-y

[64] Naranjo-Saltos F, Hallo A, Hallo C, Mayancela A, Rojas A. Gastrointestinal cryptococcosis associated with intestinal lymphangiectasia. Case Rep Med. 2020;2020:7870154. 32373179 10.1155/2020/7870154 PMC7191395

[65] Lee SJ, Song HJ, Boo SJ, Na SY, Kim HU, Hyun CL. Primary intestinal lymphangiectasia with generalized warts. World J Gastroenterol. 2015;21:8467–72. 26217101 10.3748/wjg.v21.i27.8467 PMC4507119

[66] Ward M, Le Roux A, Small WP, Sircus W. Malignant lymphoma and extensive viral wart formation in a patient with intestinal lymphangiectasia and lymphocyte depletion. Postgrad Med J. 1977;53:753–7. 604991 10.1136/pgmj.53.626.753 PMC2496800

[67] Dierselhuis MP, Boelens JJ, Versteegh FG, Weemaes C, Wulffraat NM. Recurrent and opportunistic infections in children with primary intestinal lymphangiectasia. J Pediatr Gastroenterol Nutr. 2007;44:382–5. 17325562 10.1097/01.mpg.0000233192.77521.2f

[68] Marie I, Lévesque H, Héron F, Courtois H, Callat MP. Gelatinous transformation of the bone marrow: an uncommon manifestation of intestinal lymphangiectasia (Waldmann’s disease). Am J Med. 1999;107:99–100. 10403358 10.1016/s0002-9343(99)00035-2

[69] Bogdanović R, Stanković I, Jojić N, Ognjanović M, Zlatković M, Popović O, et al. Recurrent hemolytic uremic syndrome with hypocomplementemia and intestinal lymphangiectasia. Nephron. 1997;76:481–4. 9274848 10.1159/000190232

[70] Kalman S, Bakkaloğlu S, Dalgiç B, Ozkaya O, Söylemezoğlu O, Buyan N. Recurrent hemolytic uremic syndrome associated with intestinal lymphangiectasia. J Nephrol. 2007;20:246–9. 17514630

[71] Milazzo L, Peri AM, Lodi L, Gubertini G, Ridolfo AL, Antinori S. Intestinal lymphangiectasia and reversible high liver stiffness. Hepatology. 2014;60:759–61. 24449480 10.1002/hep.27025

[72] Licinio R, Principi M, Ierardi E, Di Leo A. Liver fibrosis in primary intestinal lymphangiectasia: an undervalued topic. World J Hepatol. 2014;6:685–7. 25276285 10.4254/wjh.v6.i9.685 PMC4179148

[73] Sahli H, Ben Mbarek R, Elleuch M, Azzouz D, Meddeb N, Chéour E, et al. Osteomalacia in a patient with primary intestinal lymphangiectasis (Waldmann’s disease). Joint Bone Spine. 2008;75:73–5. 17900962 10.1016/j.jbspin.2007.01.045

[74] Holt PR. Dietary treatment of protein loss in intestinal lymphangiectasia. The effect of eliminating dietary long chain triglycerides on albumin metabolism in this condition. Pediatrics. 1964;34:629–35. 14227642

[75] Jadhav HB, Annapure US. Triglycerides of medium-chain fatty acids: a concise review. J Food Sci Technol. 2023;60:2143–52. 35761969 10.1007/s13197-022-05499-w PMC9217113

[76] Alfano V, Tritto G, Alfonsi L, Cella A, Pasanisi F, Contaldo F. Stable reversal of pathologic signs of primitive intestinal lymphangiectasia with a hypolipidic, MCT-enriched diet. Nutrition. 2000;16:303–4. 10758368 10.1016/s0899-9007(00)00223-9

[77] Aoyagi K, Iida M, Matsumoto T, Sakisaka S. Enteral nutrition as a primary therapy for intestinal lymphangiectasia: value of elemental diet and polymeric diet compared with total parenteral nutrition. Dig Dis Sci. 2005;50:1467–70. 16110837 10.1007/s10620-005-2863-7

[78] Bredefeld C, Hussain MM, Averna M, Black DD, Brin MF, Burnett JR, Charrière S, et al. Guidance for the diagnosis and treatment of hypolipidemia disorders. J Clin Lipidol. 2022;16:797–812. 36243606 10.1016/j.jacl.2022.08.009

[79] Ballinger AB, Farthing MJ. Octreotide in the treatment of intestinal lymphangiectasia. Eur J Gastroenterol Hepatol. 1998;10:699–702. 9744700

[80] Sari S, Baris Z, Dalgic B. Primary intestinal lymphangiectasia in children: is octreotide an effective and safe option in the treatment? J Pediatr Gastroenterol Nutr. 2010;51:454–7. 20512058 10.1097/MPG.0b013e3181d1b162

[81] Al Sinani S, Rawahi YA, Abdoon H. Octreotide in Hennekam syndrome-associated intestinal lymphangiectasia. World J Gastroenterol. 2012;18:6333–7. 23180957 10.3748/wjg.v18.i43.6333 PMC3501785

[82] Altit G, Patel H, Morinville VD. Octreotide management of intestinal lymphangiectasia in a teenage heart transplant patient. J Pediatr Gastroenterol Nutr. 2012;54:824–7. 21768882 10.1097/MPG.0b013e31822d2dd4

[83] Hammill AM, Wentzel M, Gupta A, Nelson S, Lucky A, Elluru R, et al. Sirolimus for the treatment of complicated vascular anomalies in children. Pediatr Blood Cancer. 2011;57:1018–24. 21445948 10.1002/pbc.23124

[84] Adams DM, Trenor CC 3rd, Hammill AM, Vinks AA, Patel MN, Chaudry G, et al. Efficacy and safety of sirolimus in the treatment of complicated vascular anomalies. Pediatrics. 2016;137: e20153257. 26783326 10.1542/peds.2015-3257 PMC4732362

[85] Ingle GR, Sievers TM, Holt CD. Sirolimus: continuing the evolution of transplant immunosuppression. Ann Pharmacother. 2000;34:1044–55. 10981252 10.1345/aph.19380

[86] Kwon Y, Kim ES, Choe YH, Kim MJ. Individual approach for treatment of primary intestinal lymphangiectasia in children: single-center experience and review of the literature. BMC Pediatr. 2021;21:21. 33407260 10.1186/s12887-020-02447-5 PMC7789338

[87] Nadal M, Giraudeau B, Tavernier E, Jonville-Bera AP, Lorette G, Maruani A. Efficacy and safety of mammalian target of rapamycin inhibitors in vascular anomalies: a systematic review. Acta Derm Venereol. 2016;96:448–52. 26607948 10.2340/00015555-2300

[88] Ozeki M, Hori T, Kanda K, Kawamoto N, Ibuka T, Miyazaki T, et al. Everolimus for primary intestinal lymphangiectasia with protein-losing enteropathy. Pediatrics. 2016;137: e20152562. 26908672 10.1542/peds.2015-2562

[89] Liviskie CJ, Brennan CC, McPherson CC, Vesoulis ZA. Propranolol for the treatment of lymphatic malformations in a neonate—a case report and review of literature. J Pediatr Pharmacol Ther. 2020;25:155–62. 32071591 10.5863/1551-6776-25.2.155 PMC7025746

[90] Poralla C, Specht S, Born M, Müller A, Bartmann P, Müller A. Treatment of congenital generalized lymphangiectasia with propranolol in a preterm infant. Pediatrics. 2014;133:e439–42. 24446440 10.1542/peds.2012-2087

[91] Persic M, Browse NL, Prpic I. Intestinal lymphangiectasia and protein losing enteropathy responding to small bowel resection. Arch Dis Child. 1998;78:194. 9579169 10.1136/adc.78.2.194 PMC1717458

[92] Kim NR, Lee SK, Suh YL. Primary intestinal lymphangiectasia successfully treated by segmental resections of small bowel. J Pediatr Surg. 2009;44:e13–7. 19853733 10.1016/j.jpedsurg.2009.06.034

[93] Kwon Y, Kim ES, Choe YH, Hyun D, Kim MJ. Therapeutic lymphatic embolization in pediatric primary intestinal lymphangiectasia. Yonsei Med J. 2021;62:470–3. 33908219 10.3349/ymj.2021.62.5.470 PMC8084696

[94] Valdes L, Huggins JT, Gude F, Ferreiro L, Alvarez-Dobano JM, Golpe A, et al. Characteristics of patients with yellow nail syndrome and pleural effusion. Respirology. 2014;19:985–92. 25123563 10.1111/resp.12357

[95] Mummadi S, Kumbam A, Hahn P. Malignant pleural effusions and the role of talc poudrage and talc slurry: a systematic review and meta-analysis. F1000Res. 2014;3:254. 25878773 10.12688/f1000research.5538.1 PMC4382843

[96] Dipper A, Jones HE, Bhatnagar R, Preston NJ, Maskell N, Clive AO. Interventions for the management of malignant pleural effusions: a network meta-analysis. Cochrane Database Syst Rev. 2020;4(4):CD010529. 32315458 10.1002/14651858.CD010529.pub3 PMC7173736

[97] Vignes S, Albuisson J, Champion L, Constans J, Tauveron V, Malloizel J, et al. French National Referral Center for Primary Lymphedema. Primary lymphedema French National Diagnosis and Care Protocol (PNDS; Protocole National de Diagnostic et de Soins). Orphanet J Rare Dis. 2021;16:18. 33407666 10.1186/s13023-020-01652-w PMC7789008

[98] Vignes S, Poizeau F, Dupuy A. Cellulitis risk factors for patients with primary or secondary lymphedema. J Vasc Surg Venous Lymphat Disord. 2022;10:179-85.e1. 33957278 10.1016/j.jvsv.2021.04.009

[99] Quemere MP, Descargues G, Verspyck E, Marpeau L. Maladie de Waldmann et grossesse. J Gynecol Obstet Biol Reprod. 2000;29:517–9. 11011282

