# Supplementary material for: Primary intestinal lymphangiectasia: French National Diagnosis and Care Protocol (PNDS; Protocole National de Diagnostic et de Soins)
Source: Orphanet J Rare Dis. 2025 Jul 10;20:356. doi: 10.1186/s13023-025-03657-9 (PMC12247264; doi:10.1186/s13023-025-03657-9)
Supplement: Supplementary file 1 — Additional file1 (DOCX 19 kb) [file 13023_2025_3657_MOESM1_ESM.docx]

**Appendix 1.**

**Fact sheet on the low-fat diet for children: low in long-chain fatty acids (LFAs) and enriched with medium-chain triglycerides (MCTs)**

Fats containing LFAs are the most common in our daily diet. Compliance with this diet is low when difficulties understanding it persist in a precarious psychosocial context, requiring regular dietary monitoring. Psychological follow-up is often necessary to maintain this strict diet over time (highly restrictive diet). This exclusion diet must be rigorously adhered to during the first years of the disease and can then be gradually extended according to the child's clinical and biological tolerance.

**Diet principles**

- Strictly low-fat diet: lipids <10% TEI*: nutritional education

- MCT-enriched: to ensure lipid requirements are met

- High-protein diet

- Cover EFA requirements (see table below)

- Ensure energy requirements are met.

**Age-related essential fatty acid requirements**

| **Age, years** | **Linoleic acid** | **Alpha-linolenic acid** | Docosahexaenoic acid (DHA) |
| --- | --- | --- | --- |
| 0–3 | 2.7% TEI | 0.45% TEI | 70 mg/d |
| 3–9 | 4% TEI | 1% TEI | 125 mg/d |
| 10–18 | 4% TEI | 1% TEI | 250 mg/d |

*TEI: total energy intake

**Specific dietetic products**

➔ Infant milk substitutes: nutritionally complete powdered formulas, rich in medium-chain triglycerides (MCTs) and low in long-chain fatty acid (LFAs), from birth.

➔ Use for as long as possible even beyond 3 years.

**Fat substitutes**

These products are essential to meet lipid requirements. They are exclusively EFA-free MCTs. Coconut oil/coconut fat: naturally MCT-containing (60%. Although it is less recommended pure because it contains 25% long-chain triglycerides, it is the basic ingredient (with palm oil) of specific MCT-rich products. It can be used in developing countries without access to products of substitution.

Only specific infant-milk formulas (enriched with EFAs), vegetable oils, fatty fish or EFA capsules can meet EFA requirements.

**Therapeutic nutritional education**

An experienced dietician is required to explain this restrictive diet, which is as selective as it is restrictive. For each food group, patients and/or their parents need to know which foods have lowest fat contents. Patients and/or parents must learn how to read food labels.

**Appendix 2. Lists of recommended and not recommended foods**

| **Foodstuff** | **Recommended** | **Not recommended** |
| --- | --- | --- |
| **Dairy products** | Skim milk  Vegetable milks (rice, chestnuts, buckwheat labeled “rich in calcium”)  Skim or partially skimmed milk yogurt  Low-fat (0%) cottage or farmer’s cheese  0% fat cream | Whole milk, concentrated milk, 2% fat milk  Whole milk yogurt  >20%/40% fat cottage or farmer’s cheese  Ice cream |
| **Meat** | Skinless chicken breast, turkey, veal | All |
| **Offal** | None | All |
| **Delicatessen meats** | Turkey breast  Air/sun-dried meat (jerky)  Sausage with 13% fat (maximum intake: 25 g)  Canadian bacon 2% fat (maximum intake: 100 g/day) | All other delicatessen meats |
| **Fish** | Fresh and frozen raw fish: bass, cod and trout (maximum intake: 100 g/day)  Pike, sole, plaice, sea bream, haddock, whiting, salt cod, perch, ray, black or yellow pollack, monkfish, squid, bacore fresh or canned | Smoked, breaded, canned in oil or white wine, in a sauce or fried  Salmon, herring, mackerel, sardines, fresh tuna, eel, halibut, turbot |
| **Seafood** | Pink shrimp, mussels, steamed scallops, whelks/sea snails, non-milky oysters, periwinkle marine snails, squid, crab, lobster | All other crustaceans and shell fish |
| **Eggs** | Hard-boiled egg whites | Egg yolk |
| **Starches/Breads** | Rice, pasta, semolina, potatoes, quinoa  Cereals: corn flakes, frosted flakes  Dried beans, lentils  Canned corn (maximum intake: 200 g/day), red (kidney) beans, canned peas, coral lentils, cooked lentils (maximum intake: 100 g/day)  White bread, wheat flake rye bread, rice cakes | Chickpeas, dry yellow soybeans, whole green soybeans, Jerusalem artichoke, fried potatoes, raviolis, cannelloni …  Cereal-rich bread, corn bread, oatmeal, white bread, pastries, biscuits, oat flour, soybean flour, salted popcorn, whole grain rice, muesli |
| **Vegetables** | All fresh, frozen green vegetables (according to personal tolerance) or canned unseasoned | Commercial vegetable soup with cream: creamed mushroom, creamed asparagus, bean sprouts |
| **Fruits** | All fresh fruit, in syrup, stewed EXCEPT fresh chestnuts (maximum intake: 100 g/day)  Dried fruits: apricots, raisins, dates, figs (maximum intake: 100 g/day) | Oleaginous fruits: walnuts, hazelnuts, almonds, cashews, coconut, pine nuts, pistachios, peanuts, olives, avocados |
| **Fats** | MCT oil used in salad dressing, mayonnaise (1 egg yolk authorized for the allowed MCT oil dose  Crème fraiche with 5% fat content (3 soupspoons/day)  Margarine with 15% fat content (1 teaspoon/day) | All cooked or raw fats (oil, butter) |
| **Sweets** | Sugar, jams/jellies, molasses, honey, chocolate (cacao powder) for breakfast, chestnut cream, fruit jelly candies, sherberts (maximum intake: 2 scoops/day)  Very low-fat content biscuits, spice cake (2/day) | Nougat, almond paste (marzipan).  All other biscuits |
| **Beverages** | Flat or sparkling water, fruit juices, tisanes, moderate amounts of tea and coffee | Alcoholic beverages |
| **Herbs and spices** | Herbs, spices, vinegar, pickles, mustard, Ketchup | All grains (sesame, pumpkin, squash, poppyseeds …) |
